# Abnormal mitochondrial transport and morphology as early pathological changes in human models of spinal muscular atrophy

**DOI:** 10.1242/dmm.021766

**Published:** 2016-01-01

**Authors:** Chong-Chong Xu, Kyle R. Denton, Zhi-Bo Wang, Xiaoqing Zhang, Xue-Jun Li

**Affiliations:** 1 Department of Neuroscience, University of Connecticut Health Center, Farmington, CT 06030, USA; 2 Department of Regenerative Medicine, Tongji University School of Medicine, Shanghai 200092, China; 3 Stem Cell Institute, University of Connecticut, Farmington, CT 06030, USA

**Keywords:** Induced pluripotent stem cells, iPSCs, Human embryonic stem cells, hESCs, Spinal muscular atrophy, Mitochondrial transport and morphology

## Abstract

Spinal muscular atrophy (SMA), characterized by specific degeneration of spinal motor neurons, is caused by mutations in the survival of motor neuron 1, telomeric (*SMN1*) gene and subsequent decreased levels of functional SMN. How the deficiency of SMN, a ubiquitously expressed protein, leads to spinal motor neuron-specific degeneration in individuals affected by SMA remains unknown. In this study, we examined the role of SMN in mitochondrial axonal transport and morphology in human motor neurons by generating SMA type 1 patient-specific induced pluripotent stem cells (iPSCs) and differentiating these cells into spinal motor neurons. The initial specification of spinal motor neurons was not affected, but these SMA spinal motor neurons specifically degenerated following long-term culture. Moreover, at an early stage in SMA spinal motor neurons, but not in SMA forebrain neurons, the number of mitochondria, mitochondrial area and mitochondrial transport were significantly reduced in axons. Knocking down of *SMN* expression led to similar mitochondrial defects in spinal motor neurons derived from human embryonic stem cells, confirming that SMN deficiency results in impaired mitochondrial dynamics. Finally, the application of *N*-acetylcysteine (NAC) mitigated the impairment in mitochondrial transport and morphology and rescued motor neuron degeneration in SMA long-term cultures. Furthermore, NAC ameliorated the reduction in mitochondrial membrane potential in SMA spinal motor neurons, suggesting that NAC might rescue apoptosis and motor neuron degeneration by improving mitochondrial health. Overall, our data demonstrate that SMN deficiency results in abnormal mitochondrial transport and morphology and a subsequent reduction in mitochondrial health, which are implicated in the specific degeneration of spinal motor neurons in SMA.

## Abstract (precis)

**Summary:** This study provides the first evidence in human models of spinal muscular atrophy of impaired mitochondrial dynamics, which serve as potential therapeutic targets for this devastating disease.

## INTRODUCTION

Spinal muscular atrophy (SMA), the leading genetic cause of death in infants and toddlers, is characterized by spinal motor neuron-specific degeneration and subsequent muscle weakness and paralysis (Pearn, 1978, 1980). This devastating disease is caused by homologous deletion or mutations of the survival of motor neuron 1, telomeric (*SMN1*) gene, leading to decreased levels of functional SMN protein (Burglen et al., 1996; Lefebvre et al., 1995). Although SMN protein is ubiquitously distributed, a reduction of functional SMN has a profound effect on spinal motor neurons, leading to specific degeneration of these cells in individuals affected by SMA. The prominent pathophysiological changes in SMA are axonal and neuromuscular junction abnormalities (Jablonka et al., 2004; Kong et al., 2009; McWhorter et al., 2003; Rossoll et al., 2003). How axonal and synaptic functions are affected and why motor neurons specifically degenerate in SMA remain largely unclear. SMN protein, which has a housekeeping role in mRNA splicing, is concentrated in discrete foci called gems in the nucleus of many cell types (Akten et al., 2011; Zhang et al., 2006). In addition to its nuclear location, SMN protein is also present in the dendrites and axons of neurons and is associated with microtubules (Fan and Simard, 2002; Zhang et al., 2003). SMN is important for the assembly of axonal messenger ribonucleoprotein complexes and can interact with a variety of mRNA-binding proteins in neurons (Akten et al., 2011; Fallini et al., 2014; Kanai et al., 2004; Liu and Dreyfuss, 1996; Rossoll et al., 2002), which might affect axonal transport or the local translation of mRNA at synapses.

A recent study reported the dysfunction of mitochondria in mouse NSC-34 cells whose SMN expression was knocked down using small interfering RNA, suggesting that SMN is important for mitochondrial function (Acsadi et al., 2009). The depletion of mitochondrial DNA (Berger et al., 2003; Ripolone et al., 2015) and increased oxidative stress (Hayashi et al., 2002) have also been reported in SMA-affected individuals. Our previous study (Wang et al., 2013) showed that the production of mitochondrial superoxide is significantly increased in spinal motor neurons, but not in forebrain neurons, derived from SMN-knockdown human embryonic stem cells (hESCs). Considering that spinal motor neurons are large cells that have a high energy demand, impaired mitochondrial function might be involved in the axonal defects and specific motor neuron degeneration in SMA. Spinal motor neurons have long axons, and the axonal transport of mitochondria is important for the synthesis of ATP in areas of axoplasm distant from the cell body. Reduced mitochondrial axonal transport has been observed in spinal motor neurons isolated from mouse models of amyotrophic lateral sclerosis (ALS; De Vos et al., 2007; Magrane et al., 2014), a motor neuron disease characterized by axonal degeneration. Interestingly, the defects of retrograde mitochondrial axonal transport were observed at an early stage, before the onset of the symptoms (Magrane et al., 2014), implicating the abnormal mitochondrial transport in axonal degeneration of spinal motor neurons in ALS. Similar to ALS, axonal degeneration is a common pathology in SMA, but the age of onset of SMA is much earlier. Whether the transport and morphology of mitochondria are impaired in SMA motor neurons and whether they play any role in motor neuron degeneration in SMA remain unknown.

Human pluripotent stem cells (Takahashi et al., 2007; Thomson et al., 1998; Yu et al., 2007), which have the capacity to generate all types of cells in the body, including spinal motor neurons, provide a unique source to researchers for studying the specific cell types that are affected by various diseases *in vitro*. Human models of SMA have been established by generating induced pluripotent stem cells (iPSCs) from individuals with SMA (Chang et al., 2011; Corti et al., 2012; Ebert et al., 2009; Sareen et al., 2012) and knocking down the functional SMN in hESCs (Wang et al., 2013), which recapitulate disease-specific degeneration in motor neurons. Here, using both iPSC- and hESC-based SMA models, we examined the mitochondrial axonal transport and morphology in these stem cell-derived neurons. Our data reveal a significant reduction of mitochondrial transport, numbers and size in axons of SMA spinal motor neurons, but not forebrain neurons, at an early stage, before motor neurons degenerate. Motor neurons derived from SMN-knockdown hESCs exhibit similar abnormal mitochondrial dynamics to those from SMA iPSCs, confirming the direct link between these mitochondrial defects and *SMN* deficiency. Moreover, application of *N*-acetylcysteine (NAC), which ameliorates the mitochondrial defects, also rescues the specific motor neuron degeneration, suggesting that mitochondrial defects underlie the motor neuron-specific degeneration in human SMA models.

## RESULTS

### Characterization and neural differentiation of control and SMA iPSC lines

**Fig. 1.**
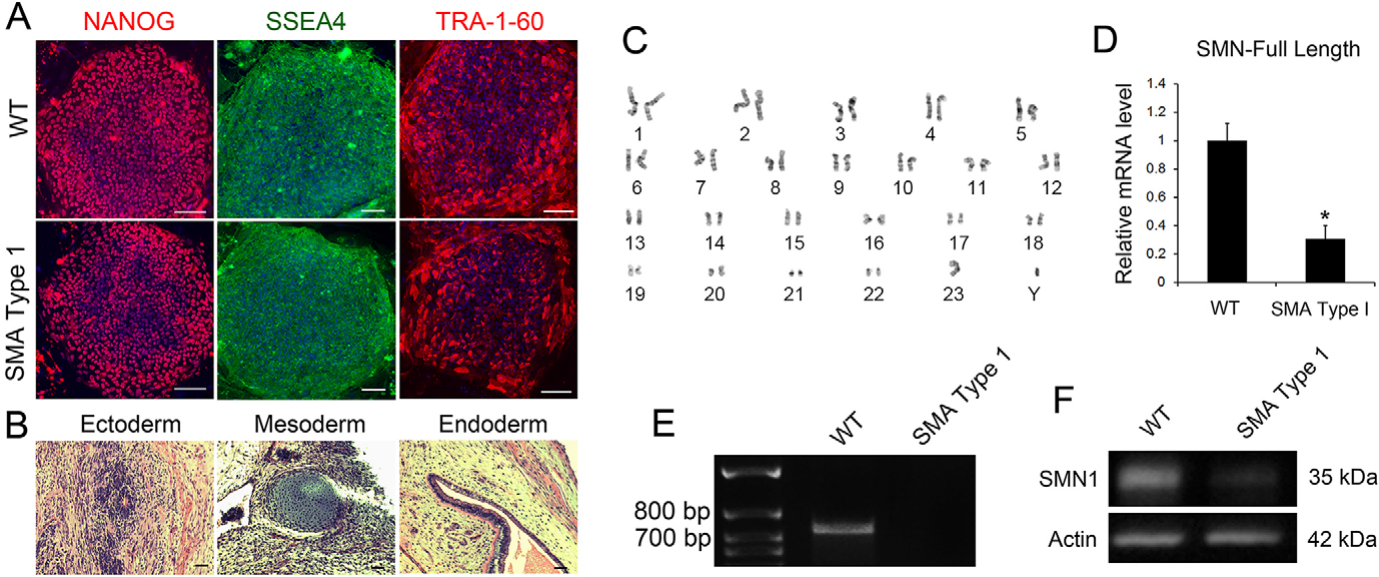
**Generation and characterization of WT and SMA iPSCs.** (A) WT and SMA type 1 iPSC lines expressed the pluripotency markers NANOG, SSEA4 and TRA-1-60. Blue indicates Hoechst-stained nuclei. Scale bars: 100 μm. (B) Hematoxylin and eosin staining of teratoma sections that were derived from iPSCs. Tissues from each germ layer were formed. Scale bars: 50 μm. (C) The SMA iPSC lines maintained a normal 46, XY male karyotype after 10 passages as shown by G-banded analysis. (D) qPCR analysis revealed a significantly decreased expression of SMN-FL mRNA in SMA type 1 iPSC lines, *n*=3. (E) PCR analysis showed the absence of *SMN1* gene in genomic DNA samples in SMA type 1 iPSC lines. (F) Western blot analysis revealed a significant decrease in SMN1 protein in SMA iPSCs compared with WT. Data are presented as mean±s.d. *P<0.05 versus WT.

We obtained fibroblast cells of SMA type 1 patients (Coriell Cell Repositories) and successfully generated iPSC clones using the episomal method (Okita et al., 2011). Control iPSC lines (wild type, WT) were also generated from fibroblast cells of a normal individual. The episomal vectors containing pluripotent factors are progressively lost from cells, leading to the generation of iPSCs free of vector and exogenous sequence. We then analyzed iPSC lines that were derived from both the SMA type 1 and WT fibroblast cells. These iPSC lines exhibited characteristic hESC-like morphology and expressed the pluripotency markers NANOG, TRA-1-60 and SSEA4 (Fig. 1A). In order to validate the pluripotency of the iPSC lines, we examined the formation of teratomas after injecting the iPSCs into SCID mice. Both the SMA type 1 and WT iPSC lines were pluripotent as revealed by their ability to differentiate spontaneously into tissues of each of the three germ layers (Fig. 1B). Considering that iPSCs are susceptible to chromosomal abnormalities, we then performed karyotype analysis. As shown in Fig. 1C, normal karyotyping was maintained even after multiple passages.

In order to model the disease successfully, another important criterion is the maintenance of gene mutations during reprogramming and subsequent differentiation. In SMA patient iPSCs, homologous deletion of the *SMN1* gene results in reduced levels of functional SMN. As expected, the mRNA expression of functional SMN (SMN-full length, SMN-FL) was significantly decreased in SMA type 1 iPSC lines compared with the WT (Fig. 1D). Using a primer set that is specific to the *SMN1* gene, we then examined the expression of *SMN1* gene in DNAs isolated from control and SMA iPSCs. RT-PCR analysis showed that *SMN1* gene was absent from SMA iPSCs, confirming the loss of *SMN1* gene (Fig. 1E). At the protein level, the expression of SMN-FL protein in SMA type 1 iPSCs was significantly decreased compared with that in WT iPSCs (∼20% of the control; Fig. 1F), confirming a reduced level of functional SMN in SMA iPSCs.

**Fig. 2.**
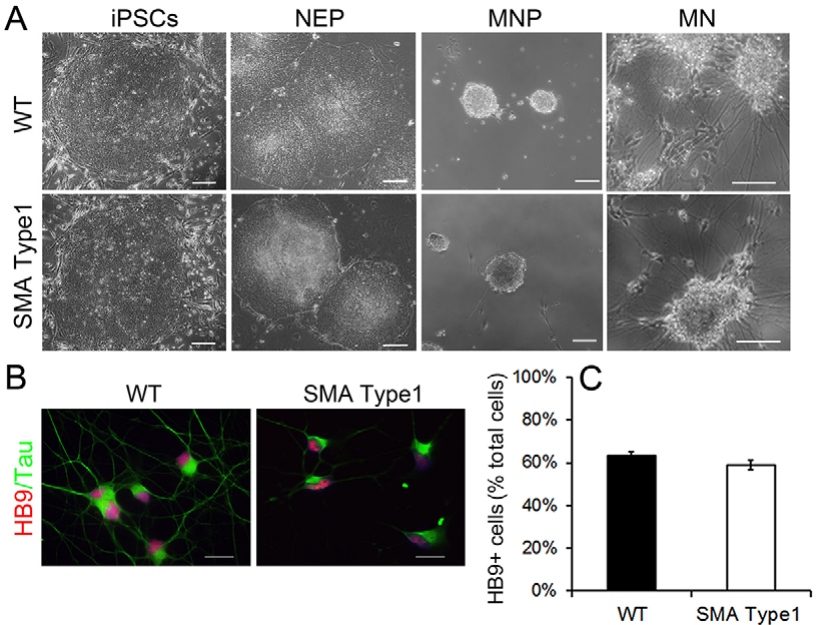
**Differentiation of spinal motor neuron from SMA** **type 1** **and WT iPSCs.** (A) Representative phase contrast images of WT control and SMA type 1 iPSCs during neural differentiation at various stages. MN, motor neurons; MNP, motor neuron progenitor; NEP, neuroepithelial progenitor. Scale bar: 100 μm. (B) Immunostaining showing the generation of HB9^+^ spinal motor neurons from WT and SMA type 1 iPSCs at day 24 after differentiation. Blue indicates Hoechst-stained nuclei. Scale bars: 20 μm. (C) Quantification of HB9^+^ neurons did not reveal any significant differences between SMA and WT groups. Data are presented as mean±s.d., *n*=6.

In order to compare the spinal motor neurons from control and SMA iPSCs, we differentiated these iPSCs into spinal motor neurons using a differentiation protocol modified from our previous methods (Li et al., 2005, 2008; Zeng et al., 2010). Human iPSCs were first differentiated to neuroepithelial cells, which were then treated with retinoic acid (RA) for caudalization and purmorphamine for ventralization (Chen et al., 2014; Du et al., 2015) (Fig. 2A). Motor neuron-enriched progenitors were isolated and suspended at 2 weeks after differentiation from iPSCs. For terminal differentiation, motor neuron-enriched clusters were dissociated and plated onto polyornithine- and laminin-coated coverslips at day 19. Five days after plating, these coverslips were fixed and subjected to immunostaining for HB9 (a marker for spinal motor neuron) and Tau (an axonal marker; Fig. 2B). The proportion of HB9^+^ postmitotic motor neurons was ∼60%, and there was no significant difference between control and SMA groups (Fig. 2C). These data suggest that spinal motor neurons can be specified efficiently from SMA iPSCs and that the initial specification of HB9^+^ spinal motor neurons from SMA iPSCs is not altered.

### SMA iPSC-derived spinal motor neurons exhibited reduced mitochondrial axonal transport

**Fig. 3.**
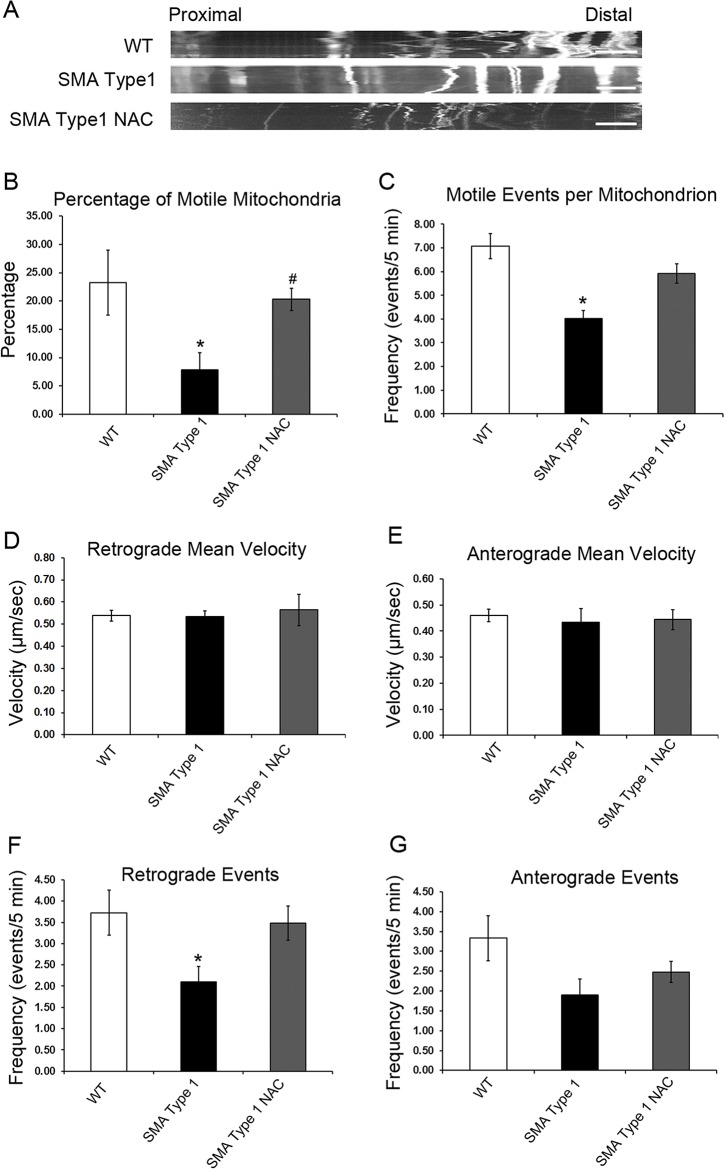
**Decreased mitochondrial axonal transport in SMA spinal motor neuron cultures.** (A) Representative distance versus time kymographs showing mitochondrial transport in day 24 spinal motor neurons. Scale bar: 10 μm. (B) The percentage of motile mitochondria was decreased in SMA type 1 spinal motor neuron cultures. The percentage of motile mitochondria was significantly increased after treatment with NAC. (C) The number of motile events per mitochondrion was also significantly decreased in SMA type 1 spinal motor neurons. (D,E) Velocities of movement events in retrograde (D) and anterograde (E) directions were not affected in SMA spinal neurons. (F,G) The frequency of motile movement events (per mitochondrion over 5 min) in the retrograde direction (F) was significantly decreased, whereas the frequency of events in the anterograde direction (G) was non-significantly reduced in SMA spinal motor neurons. Data are presented as mean±s.e.m., *n*=10-20 cells. *P<0.05 versus WT, ^#^*P*<0.05 versus SMA type 1.

SMA is characterized by axonal and synaptic defects, and recent studies have reported the dysfunction of mitochondria in SMA cell models. To understand the mechanisms underlying the functional defects of spinal motor neurons in SMA, we assessed the mitochondrial transport in SMA type 1 iPSC-derived spinal motor neuron cultures. Using the MitoTracker CMXRos dye, we first analyzed mitochondrial axonal transport in day 24 iPSC-derived neurons with live-cell imaging. As shown in Fig. 3A, representative kymographs revealed the nature of axonal transport in the neuron. The frequency of motile events was calculated by counting the number of times each mitochondrion moved with a velocity of >300 nm/s. This velocity threshold was selected to exclude actin-mediated transport events, which fall well below this threshold (De Vos and Sheetz, 2007). Calculation of the percentage of motile mitochondria for each cell revealed a significant reduction in SMA type 1 iPSC-derived spinal motor neuron cultures compared with the WT (Fig. 3B), and the frequency of motile events was also reduced in the SMA neurons (Fig. 3C).

Transport vesicles and membranous organelles can move in two different directions, i.e. from cell bodies down to the axon (anterograde transport) and from the distal part back to the cell body (retrograde transport). It is unknown whether anterograde or retrograde transport is affected in SMA. Therefore, using these SMA patient iPSC-derived spinal motor neuron cultures, we also analyzed the mitochondrial transport in both anterograde and retrograde directions. Comparison of mitochondrial transport velocities in both retrograde (Fig. 3D) and anterograde (Fig. 3E) directions showed that there were no significant differences between control and SMA neurons. There was a significant reduction in the frequency of motile events in the SMA type 1 iPSC-derived spinal motor neuron cultures compared with the WT. Further analysis of the direction of transport revealed a non-significant trend toward the reduction of events in the anterograde direction (Fig. 3G) and a significant reduction in retrograde events in SMA cells (Fig. 3F). These results suggest that mitochondrial axonal transport is impaired in SMA motor neurons at an early stage during the disease progression, which might serve as an early contributor to motor neuron degeneration in SMA.

### *N*-acetylcysteine rescued the mitochondrial transport and morphological defects

Our previous study showed that NAC, an antioxidant, could mitigate the increased mitochondrial oxidative stress caused by knocking down of SMN, leading to the rescue of motor neuron degeneration (Wang et al., 2013). In order to dissect the protective role of NAC in SMA, we examined the effect of NAC on the mitochondrial axonal transport in SMA spinal motor neuron cultures. NAC (80 μg/ml) was added to neural cultures derived from SMA iPSCs from the neural progenitor stage to the motor neuron stage (from day 13 to day 24), and mitochondrial axonal transport was examined at day 24 as described in the previous subsection. As shown in Fig. 3, application of NAC significantly increased the percentage of motile mitochondria (Fig. 3B) compared with that in SMA motor neuron cultures. These data suggest that NAC can ameliorate the mitochondrial axonal transport defects in SMA motor neurons.

**Fig. 4.**
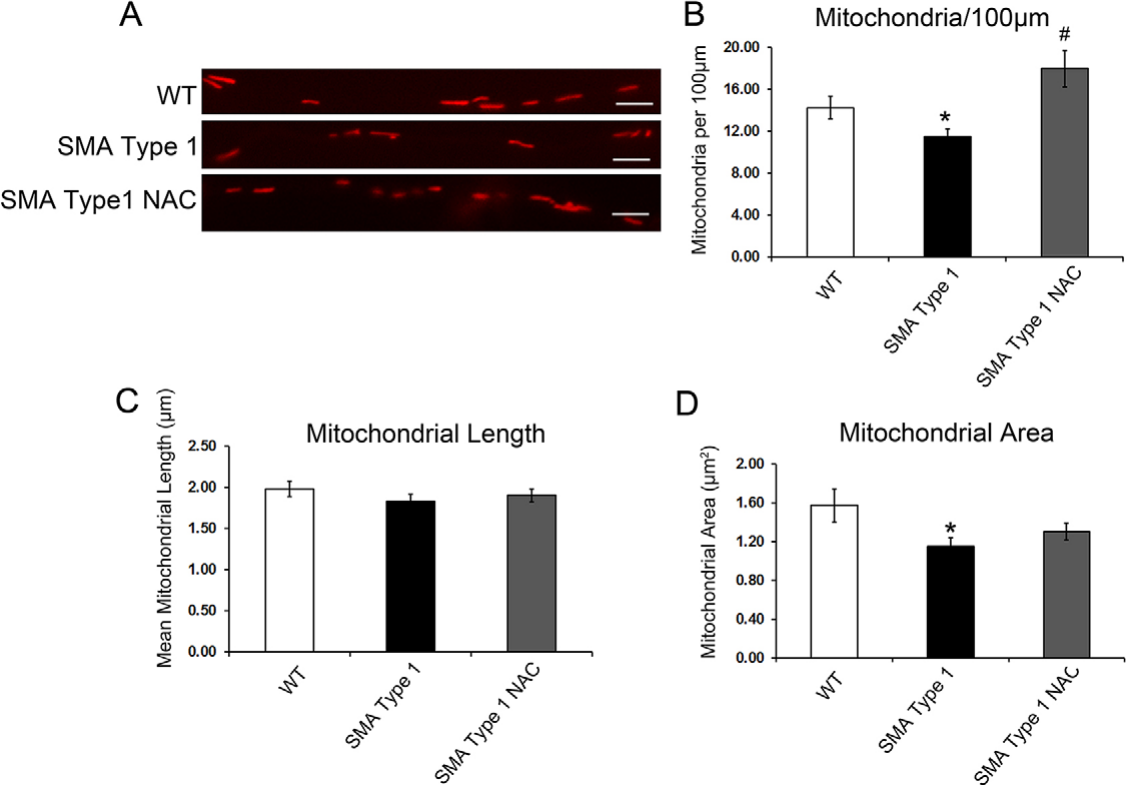
**Abnormalities of mitochondrial morphology** **in spinal motor neuron cultures.** (A) Representative images of mitochondria in day 24 spinal motor neuron axons from WT, SMA type 1 and SMA type 1 NAC-treated groups. Scale bars: 5 μm. (B) The number of mitochondria was decreased in SMA type 1-derived spinal motor neurons compared with WT. NAC, a potent antioxidant, was applied to neural cultures derived from SMA type 1 iPSCs at day 13 after differentiation. The number of mitochondria was significantly increased after treatment with NAC. (C) There were no significant differences in the length of mitochondria between different groups. (D) The mitochondrial area was significantly decreased in SMA neurons compared with WT. Data are presented as mean±s.e.m. *n*=10-20 cells. *P<0.05 versus WT, ^#^*P*<0.05 versus SMA type 1.

During the investigation of mitochondrial axonal transport *in vitro*, we constantly observed smaller mitochondria in SMA spinal motor neuron cultures compared with controls. Given that mitochondrial dynamics and distribution are important for their functions, we also analyzed the number of mitochondria and their morphology in axons of spinal motor neuron cultures at day 24. As shown in representative images of mitochondrial morphology (Fig. 4A), the number of mitochondria in SMA type 1 iPSC-derived spinal motor neuron culture was significantly decreased compared with that in the WT group (Fig. 4B). Although the average length of mitochondria was non-significantly decreased in SMA type 1 iPSC-derived spinal motor neuron cultures (Fig. 4C), the mitochondrial area was significantly decreased compared with WT (Fig. 4D). After the treatment with NAC, the number of mitochondria was significantly increased in comparison to the number in SMA motor neuron cultures (Fig. 4B). Together, our data suggest that in SMA spinal motor neurons, there are deficits in mitochondrial axonal transport, distribution and morphology, which can be partly rescued by the application of NAC.

### Knocking down SMN-FL in spinal motor neurons resulted in similar mitochondrial transport and morphology deficits

To confirm whether the abnormal mitochondrial dynamics that we observed in SMA spinal motor neuron cultures are directly linked to the deficiency of *SMN*, we examined the transport and morphology of mitochondria in spinal motor neurons derived from SMN-knockdown hESCs. In our previous study, we described the establishment of SMN-FL knockdown hESC lines that recapitulate the pathological changes in SMA (Wang et al., 2013). Using the SMN-FL RNAi and luciferase RNAi (as a control) hESCs, we then differentiated these stem cells into neural lineage and spinal motor neuron as we described for SMA iPSCs. In day 24 neurons, axonal transport of mitochondria was examined after staining with MitoTracker (Fig. S1A-G). Similar to our observations in SMA iPSC-derived cultures, we observed significant reductions in the percentage of motile mitochondria (Fig. S1B) and the frequency of motile events in the retrograde direction (Fig. S1F) in the SMN-FL-knockdown spinal motor neuron cultures compared with control luciferase RNAi cultures. The frequency of motile events showed a trend to decrease but was not statistically significant in the SMN-FL knockdown neurons (Fig. S1C). Together, the SMN-knockdown spinal motor neuron cultures showed similar reductions in the motility of mitochondria and the frequency of motile events in the retrograde direction, confirming the direct link between SMN deficiency and the mitochondrial transport deficits.

Next, we compared the number of mitochondria and the mitochondrial morphology in axons between luciferase and SMN-FL RNAi spinal motor neuron cultures (Fig. S2). As shown in representative images of mitochondrial morphology (Fig. S2A), the number of mitochondria in SMN-FL RNAi hESC-derived spinal motor neuron cultures was significantly decreased compared with that in the luciferase RNAi group (Fig. S2B). Similar to the SMA iPSC-derived motor neuron cultures, the mitochondrial area was significantly decreased in SMN-knockdown spinal motor neurons (Fig. S2D). These data confirm that loss of SMN-FL function is directly implicated in the abnormal mitochondrial transport, distribution and size in SMA spinal motor neurons. Furthermore, the application of NAC to the SMN-knockdown cultures (from day 13 to day 24) significantly mitigated the reduction of the percentage of motile mitochondria (Fig. S1B) and the number and area of mitochondria in axons (Fig. S2B,D), confirming the protective effects of NAC against mitochondrial defects in both iPSC- and hESC-based SMA models.

### Cell type-specific alterations of mitochondrial axonal transport and morphology in SMA

**Fig. 5.**
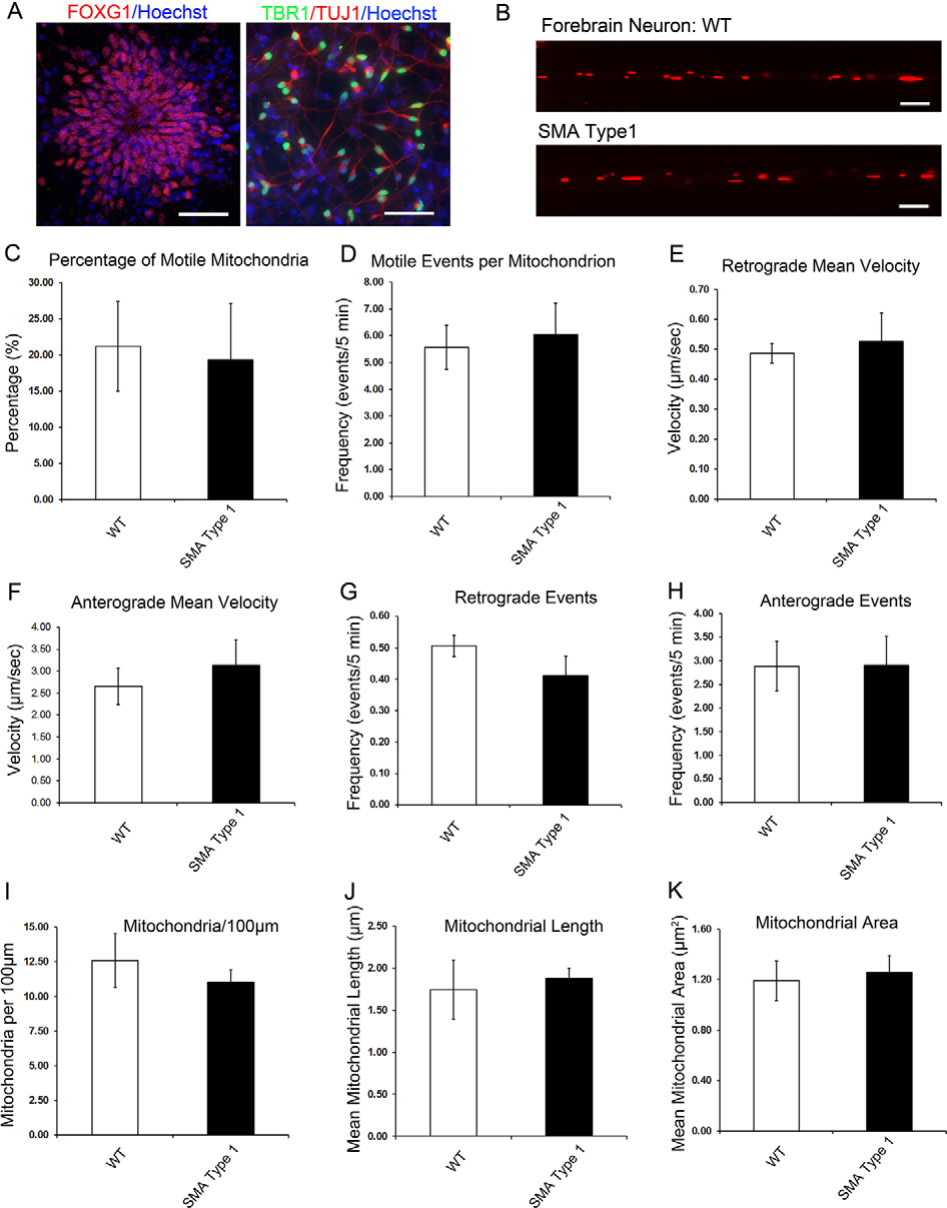
**Mitochondrial morphology and transport in SMA forebrain neuron cultures.** (A) Immunostaining shows the generation of telencephalic progenitors (FOXG1^+^) and subsequent TBR1^+^ glutamatergic forebrain neurons (TBR1^+^ cells doubled with TUJ1, a neuronal marker) from iPSCs. Scale bars: 50 μm. (B) Representative images of mitochondria in day 24 forebrain neuron axons from WT and SMA type 1 groups. Scale bars: 5 μm. (C) Quantification of the percentage of motile mitochondria. (D) Quantification of the motile events per mitochondrion (velocity >300 nm/s). (E,F) The velocities of movement events in retrograde (E) and anterograde (F) directions were not affected in SMA forebrain neuron cultures. (G,H) The frequency of motile events in the retrograde (G) and anterograde (H) directions was not altered in forebrain neuron cultures. (I) Quantification of the number of mitochondria in SMA forebrain neuron cultures. (J,K) There were no significant differences in the length (J) and area (K) of mitochondria in SMA forebrain neuron cultures between different groups. Data are presented as mean±s.e.m., *n*=10-20 cells.

Considering that spinal motor neurons specifically degenerate in SMA patients, we then tested whether the mitochondrial defects are specific to spinal motor neurons and are not observed in other types of neurons. To achieve this, we generated forebrain neurons (telencephalic glutamatergic neurons) from WT and SMA type 1 iPSCs and examined the mitochondrial axonal transport and morphology in these neurons. The iPSC lines were differentiated to forebrain neurons using a protocol we established previously, which leads to the efficient generation of telencephalic progenitors (FOXG1^+^) and subsequent TBR1^+^ glutamatergic neurons (Fig. 5A; Boisvert et al., 2013; Li et al., 2009; Zeng et al., 2010). Forebrain neurons were dissociated and plated on coverslips for terminal differentiation. At the same time point as we tested for the motor neuron cultures (day 24), the mitochondrial transport and morphology were examined and compared between WT and SMA forebrain neurons (Fig. 5B). Interestingly, there were no significant alterations in the mitochondrial axonal transport (Fig. 5C-H) and mitochondrial morphology (Fig. 5I-K) in SMA forebrain neurons compared with WT neurons. Together, these data suggest that abnormal mitochondrial dynamics in our human SMA models are specific to spinal motor neurons, which might underlie the specific degeneration of spinal motor neurons in SMA.

### Specific degeneration of spinal motor neurons in long-term cultures

**Fig. 6.**
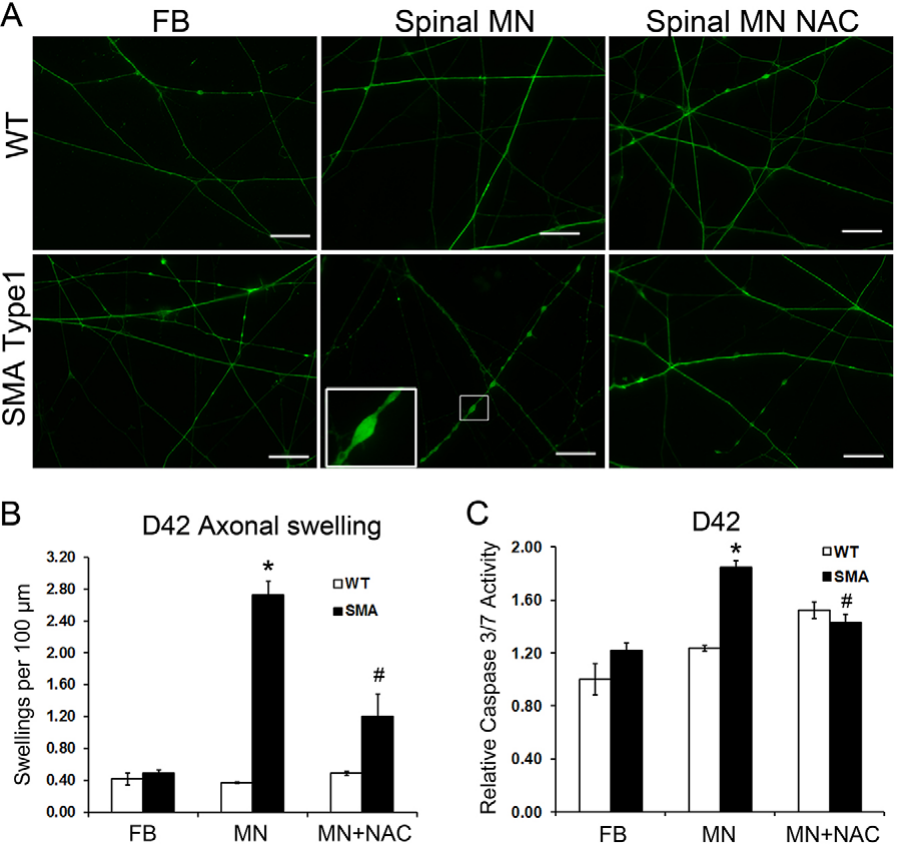
**NAC rescued the motor neuron-specific degeneration in SMA long-term cultures.** (A) Tau immunostaining shows axons of 6-week-old forebrain (FB) neuron cultures, spinal motor neuron (MN) cultures and spinal motor neuron cultures treated with NAC. Boxed areas are enlarged in insets. Scale bars: 50 μm. (B) Quantification revealed a significant increase in axonal swellings in SMA patient-derived neurons [day 42 (D42)] compared with the WT. The number of axonal swellings was significantly decreased after treatment with NAC (MN+NAC). (C) The activity of caspase 3/7 was significantly increased in motor neuron cultures (D42) derived from SMA type 1 iPSCs, and this increase was significantly inhibited by the application of NAC. Data are presented as mean±s.e.m., *n*=6-7. *P<0.05 versus WT group, ^#^*P*<0.05 versus SMA type 1 group.

A recent study reported that ALS iPSC-derived spinal motor neurons underwent degeneration and exhibited bead-like swellings along the neurites (Chen et al., 2014). Given that the SMA iPSC-derived spinal motor neurons exhibited impairment of mitochondrial axonal transport, we asked if these neurons underwent axonal degeneration, exhibited bead-like swellings and died in long-term culture. To test this, we cultured the spinal neurons on coverslips in the presence of neurotrophic factors for another 3 weeks (total 42 days after differentiation from iPSCs). Then, we performed HB9 and Tau staining to examine the formation of axonal swelling (Fig. 6A). Our data showed that the number of axonal swellings in the SMA spinal motor neurons was significantly increased compared with that in the WT spinal motor neurons (Fig. 6B). Next, in order to examine whether these neurons undergo apoptosis, we compared the caspase 3/7 activity between SMA and WT motor neuron cultures. In spinal motor neuron cultures (day 42), the activity of caspase 3/7 significantly increased in the SMA spinal motor neurons compared with that in the WT group (Fig. 6C). Interestingly, at the same time point (day 42) in forebrain neuron cultures, there were no significant differences in the number of axonal swellings (Fig. 6B) or the caspase 3/7 activities (Fig. 6C) between SMA type 1 and WT groups. These results reveal that SMA iPSC-derived spinal motor neurons specifically degenerate in long-term cultures, recapitulating the selective vulnerability in SMA.

**Fig. 7.**
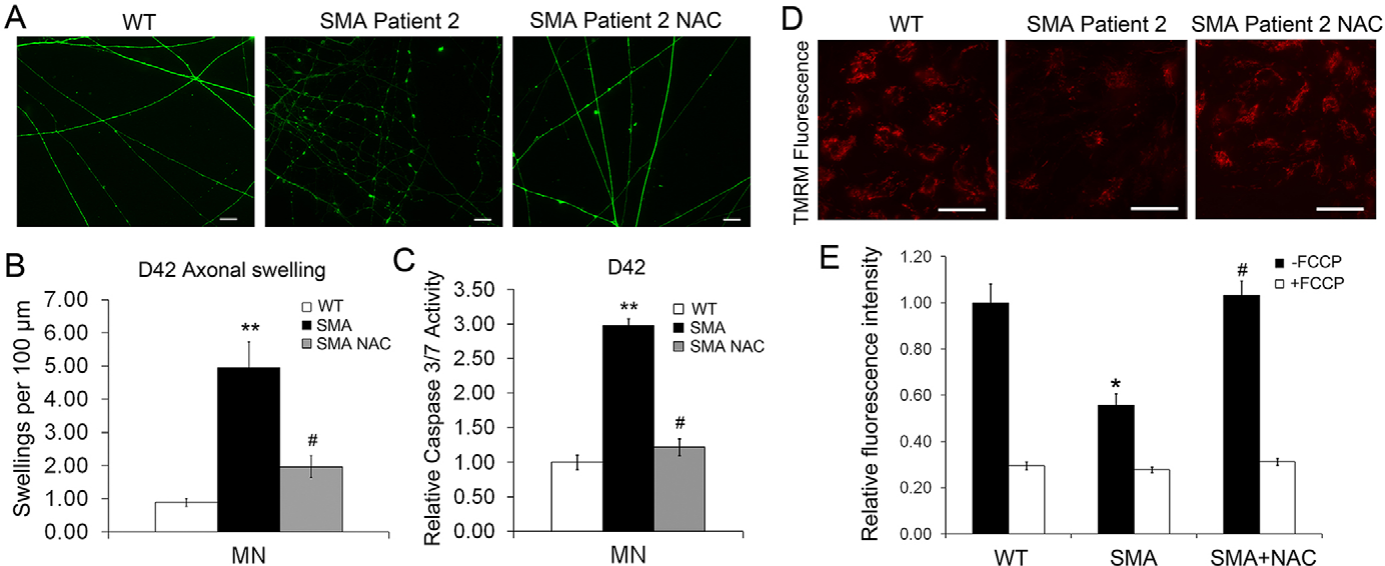
**Protective effects of NAC in spinal motor neuron cultures derived from SMA Patient 2 iPSCs.** (A) SMA iPSCs derived from a different type 1 patient (Patient 2), and control iPSCs, were differentiated into spinal motor neurons. At 6 weeks after differentiation (day 42, D42), Tau immunostaining revealed the presence of swellings along axons in SMA spinal motor neuron cultures. (B) Quantification revealed a significant increase in axonal swellings in Patient 2-derived neurons, which was mitigated by the application of NAC. Data are presented as mean±s.e.m., *n*=6 coverslips, with at least 500 axons being analyzed per group. (C) The activity of caspase 3/7 was significantly increased in SMA Patient 2 iPSC-derived motor neuron cultures (D42); the increased activity was significantly inhibited by the application of NAC. Mean±s.e.m., *n*=5. (D) To examine the mitochondrial membrane potential, the fluorescence intensity of potential-dependent dye TMRM was measured in iPSC-derived spinal motor neuron cultures (day 36) in WT, SMA Patient 2 and SMA Patient 2 NAC-treated groups. (E) Quantification revealed a significant increase in TMRM fluorescence intensity in SMA Patient 2-derived spinal motor neurons, which was rescued by the application of NAC. Data are presented as mean±s.e.m., *n*=3 coverslips, with at least 50 cells being analyzed per group. *P<0.05, **P<0.01 versus WT group, ^#^*P*<0.05 versus SMA type 1 group. Scale bars: 50 μm.

Next, we examined whether NAC, which mitigated the abnormal mitochondrial dynamics, was able to rescue the motor neuron degeneration in long-term cultures. NAC was added to SMA spinal motor neuron cultures during the same time period (from day 13 to day 42), and the formation of bead-like axonal swellings was analyzed at 42 days after differentiation (Fig. 6A). After NAC treatment, the number of axonal swellings was significantly decreased compared with SMA motor neuron cultures (Fig. 6B). Moreover, NAC ameliorated the increased caspase 3/7 activity in SMA motor neuron cultures (Fig. 6C), suggesting that NAC can rescue the specific motor neuron degeneration in long-term cultures in the SMA human model. In order to confirm the protective effect of NAC, we established iPSC lines from a second SMA patient and examined the effect of NAC in SMA Patient 2 iPSC-derived motor neurons. The SMA Patient 2 iPSC-derived motor neurons exhibited similar phenotypes, including increased axonal swellings (Fig. 7A,B) and increased apoptosis (Fig. 7C), which were also rescued by NAC (Fig. 7A-C). Together, these data reveal that application of NAC, which ameliorates the mitochondrial defects, also rescues the specific motor neuron degeneration, suggesting that mitochondrial defects underlie the motor neuron-specific degeneration in human SMA models.

Considering that alterations in mitochondrial membrane potential can result in the release of cytochrome *c* and apoptosis (Gottlieb et al., 2003), we sought to examine whether mitochondrial health was affected in SMA-derived motor neurons. The cells were incubated with the fluorescent dye TMRM, which binds to mitochondria based on the membrane potential (Perry et al., 2011). We compared the TMRM fluorescence intensity in motor neuron cultures derived from WT, SMA and SMA plus NAC-treated groups at around 5 weeks after differentiation (day 36, before motor neurons degenerated; Fig. 7D). This showed a significant reduction in TMRM fluorescence in SMA iPSC-derived motor neuron cultures compared with WT (Fig. 7E), indicating reduced mitochondrial health. The TMRM signals were significantly inhibited in all groups by applying FCCP, a mitochondrial uncoupler (Benz and McLaughlin, 1983), confirming the specificity of TMRM labeling. After NAC treatment, the TMRM fluorescence was significantly increased compared with SMA motor neuron cultures (Fig. 7D,E), indicating the improvement of mitochondrial health by NAC. Together, these data suggest that mitochondrial dysfunction, including reduced mitochondrial health, in SMA spinal motor neurons is implicated in the pathogenesis of SMA.

## DISCUSSION

Mitochondrial dysfunction has been implicated as a crucial pathological abnormality in many neurodegenerative diseases, such as ALS (Magrane et al., 2014), hereditary spastic paraplegia (Denton et al., 2014), Parkinson's disease, Alzheimer's disease and Huntington's disease (Chen and Chan, 2009). Studies have reported mitochondrial dysfunction and oxidative stress in SMA models and patients' autopsy or biopsy samples (Acsadi et al., 2009; Berger et al., 2003; Hayashi et al., 2002; Ripolone et al., 2015; Wang et al., 2013). However, the relationship between abnormal mitochondrial function and the pathogenesis of SMA remains largely unclear. SMA is characterized by axonal and synaptic defects in the spinal motor neurons. In this study, we found that the transport and density of axonal mitochondria were significantly reduced in spinal motor neurons, but not in forebrain neurons. Considering that mitochondria are important for providing energy, impaired mitochondrial axonal transport and reduced numbers in axons might be implicated in the axonal defects in SMA spinal motor neurons. Interestingly, these alterations in mitochondria happened at an early stage in motor neurons, within 1 week after the initial specification of spinal motor neurons. This suggests that SMA spinal motor neurons exhibit early defects even though the initial efficiency of differentiation is not altered. This is also in line with previous findings that the axonal outgrowth and neurite complexity are impaired in SMA motor neurons before they degenerate (Chang et al., 2011; Wang et al., 2013). Interestingly, although the mitochondrial transport in both anterograde and retrograde directions showed a trend to decrease, the retrograde transport was significantly decreased in both SMA iPSC- and SMN-knockdown hESC-derived motor neurons. Our finding agrees with a recent report on mitochondrial transport using neurons isolated from ALS mouse models, where the retrograde transport in motor neuron axons was affected first, before the onset of the symptoms (Magrane et al., 2014). The early impairment of retrograde mitochondrial transport implies its role in the motor neuron degeneration. Retrograde axonal transport is important for proper responses of neurons because it brings distal trophic factors or stress stimuli to the soma. The role of retrograde axonal transport deficits in neurodegeneration is further supported by studies showing that mutations in subunits of cytoplasmic dynein, a motor protein involved in retrograde transport, can result in motor neuron degeneration (Hafezparast et al., 2003; Puls et al., 2003). Whether the impairment of retrograde transport is specific to mitochondria or whether retrograde transport in general is affected in our SMA models needs to be investigated further.

Mitochondria undergo continuous fission and fusion to maintain normal shape and function (Chan, 2012; van der Bliek et al., 2013). Analysis of the mitochondrial morphology in SMA motor neurons reveal a significant reduction of mitochondrial density (or number) and area along axons. Alterations in mitochondrial morphology and dynamics have been previously reported in other motor neuron diseases (Magrane et al., 2014) but not in SMA. Our study provides new evidence on the involvement of abnormal mitochondrial dynamics in SMA motor neurons. Similar to the impairment of mitochondrial axonal transport, the reduction of mitochondrial density and area was also observed in motor neurons at an early stage (day 24 neurons). The reduced mitochondrial area in SMA spinal motor neurons is probably caused by the reduction of both length and width, both of which showed a trend to reduction. Although it is still not clear why the density and size of mitochondria are reduced in SMA spinal motor neurons, mitochondrial size can be affected by fission and fusion (Chan, 2012; van der Bliek et al., 2013). As shown in previous studies, increased mitochondrial fission could result in reduced mitochondrial size, leading to apoptosis (Itoh et al., 2013; Nakamura and Lipton, 2010). To examine mitochondrial health in SMA motor neuron cultures, we measured mitochondrial membrane potential using the fluorescence dye TMRM (Perry et al., 2011). Our data showed a significant reduction of TMRM fluorescence in SMA iPSC-derived spinal motor neuron cultures at around 5 weeks (day 36), indicating reduced mitochondrial health. Interestingly, this reduction was not observed in SMA cultures at early stages (day 24), when mitochondrial dynamics were impaired. Consideration that alterations in mitochondrial membrane potential can result in the release of cytochrome *c* and apoptosis (Gottlieb et al., 2003), it is possible that impaired mitochondrial dynamics might cause reduced mitochondrial health, leading to the degeneration of spinal motor neurons in SMA. In the future, it would be interesting to dissect how mitochondrial dynamics and health are affected at different stages of disease progression and how these mitochondria-related changes (transport, morphology and health) interact with each other.

SMA is caused by decreased levels of functional SMN, which is a ubiquitously expressed protein. How the decreased level of SMN leads to the mitochondrial dysfunction in SMA neurons is not clear. Nuclear SMN plays an important role in the assembly of many different classes of small ribonucleoprotein particles (snRNPs) that function in pre-mRNA splicing and gene transcription. Recently, a study reported that SMN-dependent U12 splicing events are impaired in SMA models, leading to decreased expression of a subset of transcripts that use U12 splicing (Lotti et al., 2012). This raises the possibility that the impaired mitochondrial function observed in SMA might be a direct consequence of the loss of the housekeeping role of SMN in snRNP biogenesis and pre-mRNA splicing. Another possibility lies in the role of SMN in mRNA transport in motor neuron axons. Impairment in the transport of mRNAs that are important for mitochondria and motor neurons might result in mitochondrial dysfunction, leading to motor neuron degeneration. Although the detailed mechanisms are not clear, the early alterations in mitochondrial transport and morphology suggest that mitochondria can be a potential therapeutic target for SMA. Indeed, application of NAC, an antioxidant which was previously shown to reduce the mitochondrial oxidant stress, was able to ameliorate the alterations in mitochondrial transport and morphology, improve mitochondrial health and rescue the subsequent motor neuron degeneration. The protective effects of NAC were observed in motor neuron cultures derived from two different SMA patient iPSCs and in SMN-knockdown hESCs, confirming the beneficial effects of NAC in human SMA cell models *in vitro*. In wobbler mice (Henderson et al., 1996) and animal models of ALS (Andreassen et al., 2000), administration of NAC has shown led to beneficial effects, reducing motor neuron degeneration *in vivo.* Further investigation is required to determine whether NAC or improving mitochondrial function has beneficial effects in SMA mice *in vivo* and the time window for intervention.

Selective degeneration of certain types of human neurons is fundamental to many neurodegenerative diseases, but the underlying mechanisms are not known. The development of human pluripotent stem cells, which can differentiate into various neuronal subtypes, provides a unique system to study this fundamental question. Using human pluripotent stem cell-based models of SMA, in the present study we revealed that mitochondrial deficits, including mitochondrial transport, distribution and morphology, are early pathological changes in human SMA models, which are implicated in the motor neuron-specific degeneration in SMA patients. How SMN deficiency in SMA results in mitochondrial dysfunction specifically in spinal motor neurons needs to be investigated. Better understanding of how and why mitochondrial dynamics and function are altered in SMA spinal motor neurons will provide valuable insights into identifying potential therapeutic targets for rescuing motor neuron degeneration in SMA.

## MATERIALS AND METHODS

### Reprogramming human fibroblasts into iPSC lines

Human iPSC lines were established from human fibroblasts by transfecting them with episomal plasmids (Addgene), as reported previously (Okita et al., 2011). Briefly, human fibroblasts obtained from SMA type 1 patients and normal individuals (Coriell Cell Repositories) were seeded at ∼10^5^ cells per 35 mm dish in Dulbecco's modified Eagle's medium (DMEM) supplemented with 10% fetal bovine serum (FBS) and 0.1 mM non-essential amino acids. For episomal transduction, human fibroblasts (∼500,000) were dissociated and then infected with episomal plasmids containing pluripotency factors (Oct3/4, Sox2, L-Myc, Klf4 and Lin28). At ∼1 week after electroporation transduction, cells were plated onto a 35 mm dish in DMEM supplemented with 10% FBS. After culturing for 7 days, cells were dissociated and seeded onto mouse embryonic fibroblast (MEF) feeder at ∼10^5^ cells per 100 mm dish. Two weeks later, colonies with morphologies similar to hESCs were observed. These colonies were split onto MEF feeder cells to derive iPSC lines. After several passages, homogeneous colonies with ESC-like morphology were generated. The episomal iPSCs used in this study were WT (derived from GM05659, Coriell Cell Repositories) and two SMA type 1 (from GM03813 and GM00232; Coriell Cell Repositories) lines.

For testing the formation of teratoma, about four wells of a six-well plate of iPSCs were collected, dissociated and resuspended in 50 μl hESC medium. These cells were injected, using a 1 ml U-100 insulin syringe, into the hindlimb of SCID mice (male, 4-6 weeks old). Teratomas were formed by about 2 months after the injection. After the formation of the teratoma, mice were euthanized and tumors dissected for further histological analysis. The related animal protocol was approved by the Institutional Animal Care and Use Committee.

### Motor neuron and forebrain neuron differentiation from hPSC lines

Stem cells were cultured on a feeder layer of irradiated MEFs, with the hESC media (+10 ng/ml FGF-2) changed daily. To generate spinal motor neurons from hPSCs (Chen et al., 2014; Du et al., 2015), hPSCs were first differentiated to neuroepithelia in a neural medium consisting of DMEM/F12, N2 supplement, and non-essential amino acids in the presence of SB431542 (2 μM), LDN193189 (300 nM) and CHIR99021 (3 μM) for 7 days. At day 8, the neuroepithelia were treated with RA (0.1 μM) and purmorphamine (0.5 μM) for induction of spinal motor neurons (Chen et al., 2014). For generation of forebrain neurons, RA and purmorphamine were not added. At day 14, spinal motor neuron progenitors in the form of rosettes were isolated and expanded as floating clusters in suspension in the same respective medium but without SB431542, LDN193189 and CHIR99021, for an additional 7 days before being plated on laminin substrate for the generation of mature neurons. To generate synchronized postmitotic neurons, the cultures were treated from day 18 to 21 with compound E (0.1 μM) to inhibit cell proliferation.

### Immunocytochemistry and quantification

Coverslips were fixed with 4% paraformaldehyde and immunohistochemistry was performed as previously described (Li et al., 2005). Antigen-antibody reactions were developed by appropriate fluorescence-conjugated secondary antibodies. Nuclei were visualized by Hoechst staining. Primary antibodies used in this study included mouse anti-Tra-1-60 (1:50; Santa Cruz Biotechnology), goat anti-Nanog (1:500; R&D), mouse anti-SSEA-4 (1:100; Developmental Studies Hybridoma Bank, DSHB), mouse anti-HB9 (1:50; DSHB), rabbit anti-Foxg1 (1:100; Abcam), rabbit anti-Tbr1 (1:1000; Proteintech), mouse anti-βIII-tubulin (Tuj1; 1:100; DSHB) and rabbit anti-Tau (1:200; Sigma-Aldrich). The population of HB9-expressing neurons among total differentiated cells (Hoechst labeled) was counted as described previously (Li et al., 2009). Briefly, the Zeiss microscope was used to capture images. At least four fields of each coverslip were chosen and counted using ImageJ software (National Institutes of Health). For each group, six coverslips were counted. To quantify axonal swellings, blindly selected fields were imaged from six coverslips per group. The number of axonal swellings was counted (at least 500 neurites were analyzed per group) and divided by the total length of Tau^+^ axons in each field, which were measured using ImageJ software as we described before (Denton et al., 2014).

### DNA, RNA isolation, PCR and RT-qPCR

Total RNA was extracted from cultures at different stages usingTRIzol, treated with DNase to remove genomic DNA according to the supplier's protocol (Invitrogen) and used as templates for the RT-qPCR. To examine the mRNA expression of SMN-FL, quantitative PCRs (qPCRs) were performed in a 20 μl mixture containing cDNA, primers and 1× SYBR Green PCR Master mix (Bio-Rad). Standard curves and melting curves were plotted for each set of primers to confirm that only one amplicon was generated at the same efficiency as *GAPDH*, a housekeeping gene. Expression levels of the mRNA were calculated using the comparative *C*_T_ method. The following primers were used: SMN-FL, 5′-ATGTTAATTTCATGGTACATG-3′ (forward) and 5′-GGAATGTGAGCACCTTCCTTC-3′ (reverse); and GAPDH, 5′-ATGACATCAAGAAGGTGGTG-3′ (forward) and 5′-CATACCAGGAAATGAGCTTG-3′ (reverse). To examine the expression of *SMN1* gene, DNA was isolated from iPSC cultures using a ZR Genomic DNA II Kit (Zymo Research) according to the supplier's protocol, and PCR was performed. The specific primer sequences used for *SMN1* were SMN1ex7F, 5′-TTCCTTTATTTTCCTTACAGGGTGTC-3′ and SMNex8R, 5′-CTACAACACCCTTCTCACAG-3′.

### Western blot

Cell pellets were collected and resuspended in lysis buffer with protease inhibitor cocktail (Sigma), then passed through a 28.5-gauge needle and lysed. The particulate fraction was removed by centrifugation. Proteins (10-20 μg) were separated on 10% SDS-PAGE and subjected to immunoblotting analysis. Both blocking and antibody incubations were carried out in Tris-buffered saline Tween-20 buffer [TBST; 10 mM Tris (pH 8.0), 150 mM NaCl and 0.05% Tween 20 (pH 8.0)] containing 5% non-fat dry milk. Primary antibodies used were rabbit anti-actin (1:1000; Sigma) and mouse anti-SMN1 (1:1000; Abnova). Horseradish peroxidase-conjugated secondary antibodies were detected with Western Lighting Chemiluminescence Reagent Plus (Pierce). For quantifying the SMN1 protein, SMN band intensities were normalized with actin and compared between different groups using ImageJ.

### Live cell imaging with MitoTracker

Spinal motor neuron progenitors were plated onto polyornithine- and laminin-coated 35 mm dishes (MatTek). At day 24 of total differentiation, the cells were stained with 50 nM MitoTracker Red CMXRos (Invitrogen) for 3 min to allow visualization of mitochondria and then replaced with fresh medium. Live-cell imaging was performed using a Carl Zeiss Axiovert 200M microscope equipped with an incubation chamber. Axons identified according to morphological criteria (constant thin diameter, long neurites, no branching and direct emergence from the cell body) were imaged every 5 s for 5 min, yielding 60 frames. Taking photobleaching into account, the exposure time and light intensity were carefully adjusted (∼500 ms, 25% light intensity) so that signal was not bleached by the end of 5 min of imaging time. Quantifications were performed using the same protocol as described previously (De Vos and Sheetz, 2007; Denton et al., 2014). In short, the location of each mitochondrion was manually selected using the Track Points function in MetaMorph, and parameters such as distance from cell body and velocity were recorded. A velocity threshold of 300 nm/s was used to select microtubule-based transport events (De Vos and Sheetz, 2007). Mitochondria that changed position (velocity >300 nm/s) in at least three consecutive frames were considered motile.

To analyze mitochondrial morphology, the same straightened images that were generated for measuring mitochondrial transport were used. Within ImageJ, we set the scaling of the image to match the objective used, after which the threshold function was used so that all of the mitochondria were highlighted. Next, the analyzed particles function was used with the following conditions (size=0.2-Infinity; circularity=0-1; show=Ellipses). We measured the length of each imaged axon and divided it by the number of mitochondria within the region to analyze the mitochondrial density (expressed as mitochondria per micrometer of axon). To analyze the mitochondrial area (expressed as mitochondria per square micrometer), the total mitochondrial area was measured and divided by the number of mitochondria within the region.

### Measurement of mitochondrial membrane potential

Mitochondrial membrane potential was measured based on a previous protocol (Joshi and Bakowska, 2011). Neurons were plated on 35 mm glass-bottomed dishes. The fluorescent dye tetramethylrhodamine methyl ester (TMRM; Invitrogen) was used because it accumulates in mitochondria based on mitochondrial membrane potential (Δψ_m_). Cells were washed three times with Tyrode solution containing 5 mM K^+^ and 2 mM Ca^2+^, then incubated with 10 nM TMRM in 2 ml Tyrode solution for 45 min at room temperature in the dark. Live imaging was performed using a Zeiss Axiovert 200M microscope equipped with an incubation chamber, using an EC plan-Neofluar 40×1.30 oil DIC objective. The cells were kept at 37°C in air supplemented with 5% CO_2_ while imaging. Microscope settings were optimized using control cells, and these settings were used for all other groups. Randomly selected fields were imaged every 20 s for a total of 600 s. The mitochondrial uncoupler FCCP was added to the media after 300 s, and the final concentration was 1 μM. The TMRM fluorescence intensity before and after FCCP was analyzed using Metamorph software, and at least 20 regions of interest were traced around mitochondrial structures for each cell, along with adjacent background regions. The pixel intensity for each region was determined, followed by background subtraction.

### Analysis of caspase 3/7 activity

For measurements of the activities of caspase 3 and 7, the Caspase-Glo 3/7 Assay (Promega) was carried out according to the manufacturer's instructions. Briefly, spinal motor neuron cultures were dissociated with Accutase (Invitrogen) and seeded into 96-well plates at 5000 cells per well in 50 μl of caspase-3/7 reagent. After incubation for 1 h at room temperature, luminescence from each well was then measured using a Wallac Victor2 1420 MultiLabel Counter.

### Statistical analysis

The statistical significance of mean differences among different sample groups was analyzed using Student's *t*-test. The significance level was defined as *P*<0.05.
